# Molecular pathophysiology and pharmacology of the voltage-sensing module of neuronal ion channels

**DOI:** 10.3389/fncel.2015.00259

**Published:** 2015-07-15

**Authors:** Francesco Miceli, Maria Virginia Soldovieri, Paolo Ambrosino, Michela De Maria, Laura Manocchio, Alessandro Medoro, Maurizio Taglialatela

**Affiliations:** ^1^Department of Neuroscience, University of Naples Federico IINaples, Italy; ^2^Department of Medicine and Health Sciences, University of MoliseCampobasso, Italy

**Keywords:** ion channels, voltage-sensing module, channelopathies, gating modifier, mutations

## Abstract

Voltage-gated ion channels (VGICs) are membrane proteins that switch from a closed to open state in response to changes in membrane potential, thus enabling ion fluxes across the cell membranes. The mechanism that regulate the structural rearrangements occurring in VGICs in response to changes in membrane potential still remains one of the most challenging topic of modern biophysics. Na^+^, Ca^2+^ and K^+^ voltage-gated channels are structurally formed by the assembly of four similar domains, each comprising six transmembrane segments. Each domain can be divided into two main regions: the Pore Module (PM) and the Voltage-Sensing Module (VSM). The PM (helices S_5_ and S_6_ and intervening linker) is responsible for gate opening and ion selectivity; by contrast, the VSM, comprising the first four transmembrane helices (S_1_–S_4_), undergoes the first conformational changes in response to membrane voltage variations. In particular, the S_4_ segment of each domain, which contains several positively charged residues interspersed with hydrophobic amino acids, is located within the membrane electric field and plays an essential role in voltage sensing. In neurons, specific gating properties of each channel subtype underlie a variety of biological events, ranging from the generation and propagation of electrical impulses, to the secretion of neurotransmitters and to the regulation of gene expression. Given the important functional role played by the VSM in neuronal VGICs, it is not surprising that various VSM mutations affecting the gating process of these channels are responsible for human diseases, and that compounds acting on the VSM have emerged as important investigational tools with great therapeutic potential. In the present review we will briefly describe the most recent discoveries concerning how the VSM exerts its function, how genetically inherited diseases caused by mutations occurring in the VSM affects gating in VGICs, and how several classes of drugs and toxins selectively target the VSM.

## Introduction

Voltage-dependent changes in ion fluxes are critical for the generation and propagation of electric signals in and between excitable cells. Despite extensive studies performed during the last 50 years, the mechanisms that regulate the voltage sensitivity of Voltage Gated Ion Channels (VGICs) still remain one of the most challenging topic of modern biophysics. As demonstrated by Hodgkin and Huxley (1952), voltage sensitivity is regulated by reorientation of charges (or dipoles) in response to changes in the membrane electric field. The movement of these charges has been experimentally demonstrated upon observation of voltage-activated non linear capacity currents, called *gating* or *sensing currents* (Armstrong and Bezanilla, 1973; Bezanilla et al., 1982).

Although these studies first established the biophysical basis of voltage-sensing in VGICs, a major breakthrough allowing to translate into molecular clues such theoretical background was the cloning and sequencing of the first VGIC, namely the voltage-gated Na^+^ channel (VGNC) from the electroplax of *Electrophorus electricus* (Noda et al., 1984), followed by the cloning of the first voltage-gated Ca^2+^ channel (VGCC) from rabbit skeletal muscle (Tanabe et al., 1987), and of a voltage-gated K^+^ channel (VGKC) from *Drosophila* (Papazian et al., 1987).

Subsequent studies using different techniques, including mutagenesis, fluorescence spectroscopy, and electrophysiology, have assigned specific functional roles to individual regions of VGICs; during this structure-function era, the “modular” nature of this class of membrane proteins was therefore established. More recently, characterization of the crystal structure of K_v_AP, K_v_1.2 and chimeric K_v_1.2/2.1 K^+^ channels (Jiang et al., 2003; Long et al., 2005a,b, 2007) followed by that of the NavAb VGNC from *Arcobacter butzleri* (Payandeh et al., 2011) paved the way to a detailed understanding of the intimate molecular architecture of VGIC.

In parallel, the extraordinary advancement of sequencing technologies of the last decade, have allowed the identification of numerous mutations responsible for human diseases in VGICs genes, often revealing previously unexplored functional roles of specific ion channel classes, thereby greatly expanding our understanding of the pathophysiological mechanisms underlying human diseases.

## Overall Structure of Voltage-Gated Ion Channels

**Figure 1:**
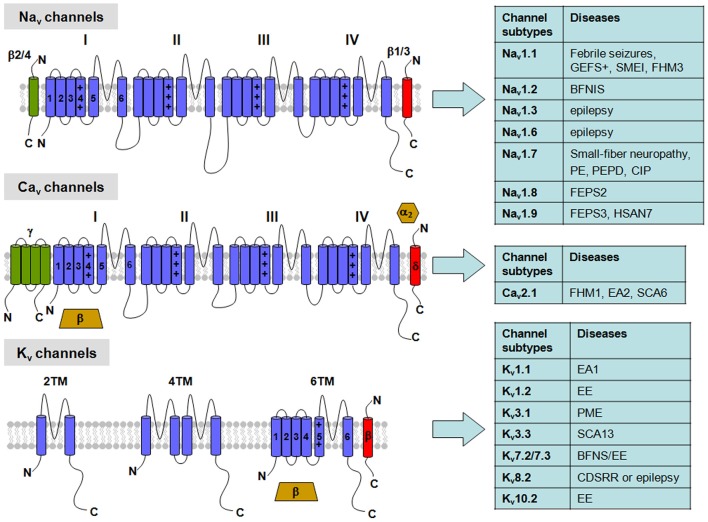
**Topological representation of voltage-gated K^+^ (K_v_), Na^+^ (Na_v_) and Ca^2+^ (Ca_v_) channels with related neuronal diseases.** Na_v_ channels are formed by a single polypeptide that contains four domains (I-IV), each with six transmembrane segments (S_1_–S_6_). β-subunits are single transmembrane proteins that co-assembles with the Na_v_ α-subunit. Ca_v_ channels show a similar topology to Na_v_ channels in their α-subunits, but can be associated with four different auxiliary subunits: the α_2_/δ-complex, linked by disulfide bridges, an intracellular β-subunit, and an occasional γ-subunit with four transmembrane segments. Abbreviations: GEFS^+^, Generalized Epilepsy with Febrile Seizures plus; SMEI, Severe Myoclonic Epilepsy of Infancy; FHM1-3, Familial Hemiplegic Migraine type 1-3, respectively; BFNS, Benign Familial Neonatal Seizures; BFNIS, Benign Familial Neonatal-Infantile Seizures; EE, Epileptic Encephalopathy; PE, Primary Erythermalgia; PEPD, Paroxysmal Extreme Pain Disorder; CIP, Congenital Insensitivity to Pain; FEPS2-3, Familial Episodic Pain Syndrome type 2-3, respectively; HSAN7, Hereditary Sensory and Autonomic Neuropathy type 7; EA1-2, Episodic Ataxia type 1-2, respectively; SCA6-13, Spinocerebellar Ataxia type 6-13, respectively; PME, Progressive Myoclonus Epilepsy; CDSRR, Cone Dystrophy with Supernormal Rod Electroretinogram.

The VGIC family represents one of the largest group of signal transduction proteins, also acting as fundamental targets for drugs with wide therapeutic applications. Primary sequence analysis indicate VGNCs and VGCCs of eukaryotes are formed by a single peptide (the α subunit) containing four homologous domains (called from I to IV). The membrane core of each of these domains contains six transmembrane helices (from S_1_ to S_6_) with an amphipathic loop between the S_5_ and S_6_ segments. By contrast, VGKCs are formed upon assembly of four compatible subunits, each showing a high sequence homology with a single domain of VGNCs or VGCCs (Figure 1). Similarly, bacterial VGNCs are composed of homotetramers of single domains whose structure resembles that of each domain of vertebrate VGNCs (Ren et al., 2001; Koishi et al., 2004), likely being the evolutionary ancestors of the larger, four-domain Na^+^ channels of eukaryotes.

In all VGICs, each structural domain can be divided into two main regions: the Pore Module (PM) and the Voltage-Sensing Module (VSM). The PM, which allows selective permeability of ion species across the cell membrane, is formed by the fifth and sixth transmembrane segments (S_5_ and S_6_, respectively) and the interconnecting loop of all subunits. The VSM of each domain senses the membrane potential variations, switching from a resting to an activated configuration, a necessary prerequisite for subsequent pore opening; the S_1_–S_4_ transmembrane segments play a crucial role in this process. Both in VGKCs (Long et al., 2007) as well as in bacterial VGNCs (Payandeh et al., 2011), the VSM of each domain interacts with the PM of a neighboring domain, thus allowing movements of the VSM module to be directly translated into conformational changes of the PM. In detail, while each of the subunits undergo voltage-dependent transitions independently (Zagotta et al., 1994), the final voltage-independent conformational change leading to pore opening appears to occur in a cooperative, all-or-none fashion (Zandany et al., 2008), suggesting only two configuration states of the pore, namely open and closed. However, this binary theory appears to not exhaustively explain the gating behavior of all VGICs: as an example, heteromeric pore conformations have been proposed for K_v_2.1 channels, as single-channel analysis revealed the occurrence of discrete subconductance levels, possibly resulting from the pore opening of only one or two subunits (Chapman and VanDongen, 2005). Whether discrete subconductance states occur and can be detected in all VGICs, and whether they result from “partial” pore opening, is still debated.

The VSM is an independent functional module that can be transplanted into other proteins to confer voltage sensitivity; indeed, voltage-sensing phosphatases (VSP) are formed by a VSM linked to a cytoplasmic phosphatase (Murata et al., 2005; Li et al., 2014c). On the other hand, the VSM can also allow ion transport by itself; indeed, subunits forming proton-selective H_v_1 channels only contain an isolated VSM, without a canonical S_5_–S_6_ pore region (Ramsey et al., 2006; Sasaki et al., 2006).

### Structural Basis of Ion Permeation and Inactivation

The PM of VGIC contains the permeation pathway that regulates ion fluxes and selectivity. Structural information regarding the molecular mechanisms of ion permeation, selectivity, and pore opening/closing were obtained from the crystal structure of bacterial “inward rectifier” K^+^ channels (K_IR_) KcsA (Doyle et al., 1998), MthK (Jiang et al., 2002a,b), and KirBac1.1 (Kuo et al., 2003), whose membrane core only contains the regions corresponding to the S_5_–S_6_ module. In these channels, the PM contains a narrow constriction near its extracellular side, which forms the selectivity filter. Below this region is a relatively large water-filled cavity at the center of the plasma membrane. In K^+^ channels, the highly conserved GYG sequence forms the selectivity filter that allows ion discrimination via direct interactions of the dehydrated K^+^ ions and the protein backbone carbonyls. While no eukaryotic VGNC structure is yet available, the crystal structure of bacterial Na_v_Ab, recently described at 2.7 Å of resolution (Payandeh et al., 2011), have revealed that a similar region is involved in ion discrimination, although Na^+^ ions permeate in their partially hydrated configuration across a wider selectivity filter. This region is likely to mediate ion discrimination also in VGNCs and VGCCs of vertebrates; in fact, it has been long known that substitution of positively charged amino acids in the PM of rat Na_v_1.2 with glutamic acid residues (which occupy equivalent positions in VGCCs), enhances Ca^2+^ ion permeability (Heinemann et al., 1992). In both VGNCs and VGCCs, a second pore-helix forms an extracellular funnel, a unique feature that could represent a conserved structural element in the outer vestibule of these channels.

Comparing the structural data of KcsA and KirBac1.1 channels, trapped in a closed conformation, with that of MthK channels, trapped in an open conformation, it has been possible to deduce structural changes that underlie pore opening in K^+^ channels. In KcsA and KirBac1.1 channels, the M2 segments, corresponding to the S_6_ segments in VGIC, form a four-helix bundle near the intracellular membrane surface that occludes the ion conduction pathway; whereas, in MthK channels the inner region of M2 is bent at a glycine residue; this residue is highly conserved among bacterial K^+^ channels and in some eukaryotic VGKCs. This glycine residue has been proposed to serve as a gating hinge that allows M2 to oscillate between a close and an open conformation. In eukaryotic K^+^ channels, it has been proposed that a conserved PVP (Proline-Valine-Proline) motif, not found in bacterial K^+^ channels and located in S_6_ downstream of the glycine residue, acts as a flexible hinge that allows channel switching from a closed to an open state (Webster et al., 2004; Long et al., 2005a,b). In particular, this inner part of the S_6_ segment, also called “bundle crossing” (BC) gate, appears to come close to the S_4_–S_5_ linker during channel activation, thus allowing to “sense” VSM movement (Prole and Yellen, 2006); BC gate widening prompts a conformational change in a further gate located at the extracellular entrance of the pore, called the “selectivity filter” gate, leading to pore opening (Labro and Snyders, 2012). The differences in single channel conductance observed between bacterial and mammalian channels might be explained by different degree of movement of the distal S_6_ regions; indeed, movements seem much wider in bacterial (having larger single channel conductance) when compared to mammalian channels, the latter having a 10-times smaller single channel conductance.

In some VGICs, currents show prominent inactivation following activation. Two main types of inactivation have been described in VGKCs: the N-type and the C-type inactivation. The N-type inactivation, which is usually more rapid than the C-type, occurs by a ball peptide tethered to the N-terminus of the channels that enters into the pore cavity blocking ion flow (Hoshi et al., 1990). By contrast, the C-type inactivation gate is located in the selectivity filter, and current inactivation is due to the pore collapse (Cuello et al., 2010a,b); structural data from bacterial KCsA confirm such a hypothesis (Bhate and McDermott, 2012). In VGNCs of vertebrates, the short intracellular loop between domains III and IV is responsible for fast inactivation by folding into the intracellular mouth of the pore and blocking it (Vassilev et al., 1988). In particular, the amino acid motif isoleucine, phenylalanine, and methionine (IFM) is required to stabilize the inactivated state of the channels (West et al., 1992). Finally, inactivation processes in VGCCs are more complex, including both voltage- and/or calcium-dependent mechanisms (Simms and Zamponi, 2014).

### Structural Basis of Voltage-Sensing

As above mentioned, voltage-sensitivity of VGICs is conferred by the VSM. In particular, the S_4_ segments are believed to play a key role in the voltage-sensitivity. Indeed, S_4_ primary sequence shows the presence of 4–8 positively-charged arginines (Rs) or lysine (Ks) residues separated by 2–3 uncharged residues; this configuration would allow positive charges to be localized on the same side of an α-helix. These basic residues are located within the electrical field existing across the two sides of the plasma membrane, thus directly sensing changes in membrane potential. The first four Rs in S_4_ are considered the most important voltage-sensing elements and are believed to be the major component of gating currents (Aggarwal and MacKinnon, 1996; Seoh et al., 1996). In K_v_1.1 channels, it has been calculated that approximately 13 elementary charges cross the electric field in response to membrane depolarization, corresponding to about three elementary charges per subunit (Schoppa et al., 1992).

The molecular mechanisms underlying VSM movement during gating are highly debated topics; nevertheless, results obtained with molecular, biochemical, electrophysiological, optic and crystallographic techniques have enormously expanded our knowledge in this field. In particular, the crystal structures of bacterial K_v_AP (Jiang et al., 2003), followed by that of mammalian K_v_1.2 and K_v_1.2/2.1 chimeric VGKCs (Long et al., 2005a, 2007), provide important information on the activated configurations of the VSM. Although no crystal structure is available for the resting state of the VSM, several hypotheses (helical-screw/sliding-helix, transporter and paddle models; see Tombola et al., 2006; Catterall, 2010; Miceli et al., 2011 for reviews) have been formulated to explain how the voltage sensor reaches its activated configuration during channel gating. Currently, these models converge toward an unified hypothesis (Khalili-Araghi et al., 2010; Vargas et al., 2011; Jensen et al., 2012; Yarov-Yarovoy et al., 2012), in which the resting and activated positions of the VSM would be stabilized by ionized hydrogen bonds between the S_4_ positive charges and two clusters of negative charges: one facing the extracellular side of the membrane and provided by the S_1_ and S_2_ helices, and another closer to the intracellular membrane surface, involving S_2_ and the proximal part of S_3_ (S_3a_) helices. At rest, S_4_ is drawn inwardly by the electrostatic forces of the negative resting membrane potential (RMP); this would allow the first S_4_ Rs (gating charges) to preferentially interact with the inner cluster of negative charges. During the activation process, S_4_ would rotate around its helical axis and translate outwardly, reaching a final configuration stabilized by the interaction of the same positively-charged residues with the negative charges provided by the extracellular cluster. This relatively small movement would allow the displacement of the gating charges from an intracellular-facing crevice to one directly connected to the extracellular milieu. A conserved phenylalanine located in the middle of S_2_ has been proposed to form the charge-transfer center that catalyzes the gating charges movement (Tao et al., 2010) and separates the extracellular and the intracellular crevices of the VSM.

In several VGKCs including K_v_1.1, the replacement of specific Rs in S_4_ with smaller amino acids widens this gating charge pathway, generating cation-permeable non-selective currents (also known as gating pore currents, or ω currents). Interestingly, gating-pore currents generated by mutations in S_4_ of Na_v_1.4 or Ca_v_1.1 channels seem to provide a pathophysiological-relevant mechanism in hypokalemic (Sokolov et al., 2007; Struyk and Cannon, 2007) or normokalemic (Sokolov et al., 2008) periodic paralysis in skeletal muscle. Similar mechanisms seem implicated in a mixed arrhythmias and the dilated cardiomyopathy caused by the R219H mutation in domain I of Na_v_1.5, which induces a proton-specific gating pore current (Gosselin-Badaroudine et al., 2012), and in peripheral nerve hyperexcitability caused by the R207Q in K_v_7.2 channels (Miceli et al., 2012). Noteworthy, sequence analysis has revealed that several other channelopathies (including some affecting neuronal channels) might be attributable to the formation of gating pore currents, however more studies are required to confirm the presence of gating pores (Moreau et al., 2014).

### Structural Basis of Electromechanical Coupling

Electromechanical coupling describes the process of transferring the energy generated by the VSM movement upon changes in membrane potential to the PM. To simplify a complex process, at resting state the S_4_–S_5_ linker directly interacts with the distal part of S_6_, making the BC gate the main voltage-controllable activation gate. Upon depolarization, S_4_ movement pulls the S_4_–S_5_ linker outwardly leading to a widening of the helical BC gate, which triggers a further conformational change at the level of the selectivity filter (SF) gate that allows ions flow through the pore. The crystal structures of K_v_1.2 and of chimeric K_v_1.2/2.1 channels suggest at least two non-covalent interactions between VSM and PM: one between the S_4_–S_5_ linker and the distal S_6_ segment at the cytoplasmic side of the membrane, and the other between the C-terminal region of S_1_ and the C-terminal region of S_5_ at extracellular site of the membrane (Lu et al., 2002; Long et al., 2005b, 2007). The interaction between S_4_–S_5_ linker and the bottom part of S_6_ seems to play an important role for transmission of conformational changes during channel gating; while the interaction between S_1_ and S_5_ acts as an anchor point between the VSM and the PM, thus allowing efficient transmission of conformational changes to the pore’s gate (Lee et al., 2009). Other interactions between VSM and PM include: the bottom part of S_4_ with S_5_ segments of neighboring subunits (Soler-Llavina et al., 2006) and the upper part of S_5_ with the upper part of S_4_ of the neighboring subunit (Lainé et al., 2003). Based on the available molecular dynamic simulations and experimental data, Blunck and Batulan (2012) proposed two different mechanisms to describe the electromechanical coupling in Shaker-like K_v_ channels. In one model S_4_ and S_4_–S_5_ linker act as a spring that is relaxed or compressed by the activation/deactivation of the VSM. In the second model, S_4_ and S_4_–S_5_ linker act as a bolt that keeps the pore closed; only when all four S_4_–S_5_ linkers have moved out the pore passively follows the opening.

The general mechanism of the electromechanical coupling seems to be conserved among the different K^+^ channels, although changes in the type of interactions and in the tightness of the coupling may occur in specific channels. Moreover, this general mechanism also seems to apply to skeletal VGNCs (Muroi et al., 2010) and VGCCs (Wall-Lacelle et al., 2011). By contrast, in hyperpolarization-activated cyclic nucleotide gated channels (HCN) decoupling between VSM and PM triggers pore closure (Blunck and Batulan, 2012).

## Neuronal Channelopathies Caused by Mutations in the VSM

### Potassium Channelopathies

#### Structure and Function of Voltage-Gated Potassium Channels (VGKCs)

**Figure 2:**
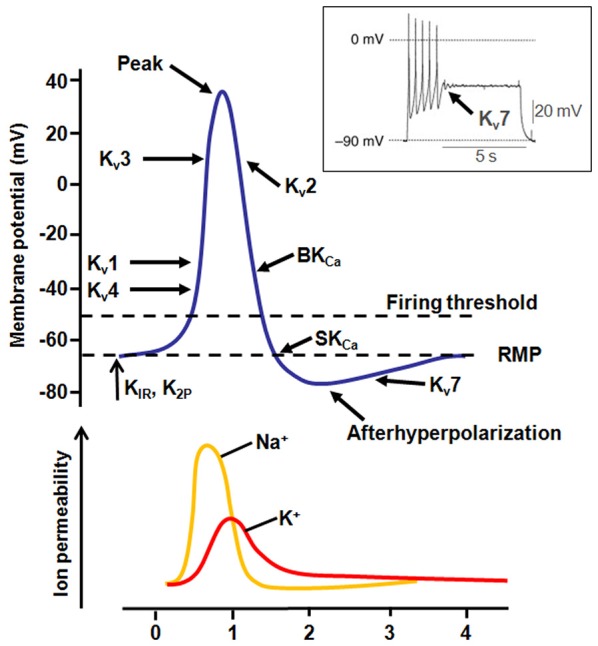
**Contribution of K^+^ currents during AP firing.** Representation of the different K^+^ channel subtypes activated during an AP. The inset shows the effect of K_v_7 channel activation during AP firing (modified from Tsantoulas and McMahon, 2014).

K^+^ channels are the largest and the most functionally heterogeneous class of ion channels expressed in all eukaryotic cells and in prokaryotes. K^+^ channels mostly inhibit neuronal excitability by setting the membrane potential closer to the K^+^ equilibrium potential. Moreover, activation of K^+^ channels shortens the duration of the action potential (AP), terminates periods of intense electrical activity, reduces neuronal firing frequency, and, in general terms, decreases the efficacy of cell excitatory inputs. Beside these roles, K^+^ channels participate in solute transport across epithelial membranes, and K^+^ clearance from brain interstitial spaces from glial cells. In humans, more than 70 genes encode for K^+^ channels. On the basis of their presumed topology, K^+^ channels can be classified into three groups: (1) the classical family with 6 transmembrane segments (6TM) including the voltage-gated K^+^ channels (K_v_ channels; K_v_1-K_v_12; Figure 1); (2) a family with only 2 transmembrane segment (2TM), homologous to the S_5_–S_6_ segments of K_v_ channels. To this group belong the inward-rectifier channels (both constitutively-active and G-protein-gated), which include at least seven gene families (K_IR_1-K_IR_7); (3) the 4 transmembrane segment family (4TM), formed by subunits encoded by at least 15 different genes (K_2P_1-K_2P_17; Yu et al., 2005). While channels formed by subunits of the first two groups are tetrameric, those of the third group are dimers. However, these three groups do not account for all K^+^ channel structural heterogeneity; in fact, large conductance Ca^2+^-dependent K^+^ channels (BK channels) assemble as tetramers of subunits containing seven transmembrane segments, which differ from K_v_ subunits for the presence of an extra transmembrane segment (S_0_) at the N-terminus.

Several VGKCs interact with accessory β-subunits that influence a wide range of channel properties such as gating, assembly, trafficking, and targeting of channels to different cellular compartments. Accessory VGKC subunits include different protein structures reflecting their divergent biological roles. Indeed, some β-subunits are integral membrane proteins such as β-subunits of both BK and K_v_7 channels; while, others are cytosolic proteins and bind to cytoplasmic modules of VGKC such as K_v_β-subunits for Shaker K_v_ channels and KChIPs for K_v_4 channels (Pongs and Schwarz, 2010 for review).

#### K_v_1.1 (*KCNA1*) and K_v_1.2 (*KCNA2*) Channel Mutations Cause Episodic Ataxia Type 1 (EA1) or Epileptic Encephalopathy (EE), Respectively

**Figure 3:**
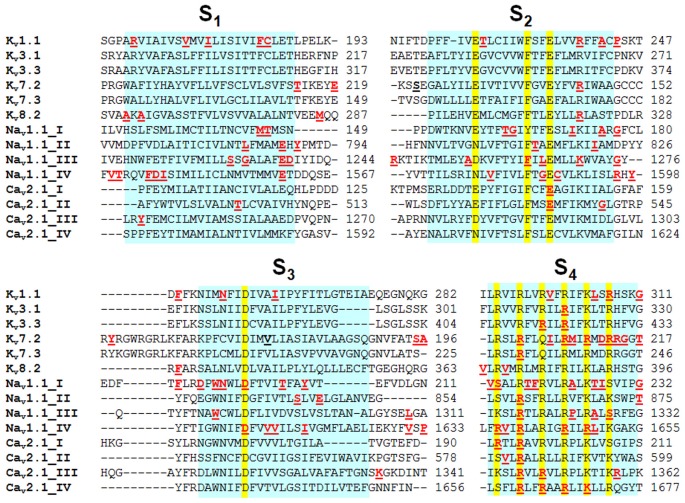
**Alignment of the VSM of different K_v_, Na_v_ and Ca_v_ channel subtypes.** The VSM have been aligned by using ClustalW2 software; highlighted in yellow are the missense mutations causing EA1 (in K_v_1.1); PME (in K_v_3.1); SCA13 (in K_v_3.3); BFNS (in K_v_7.2 and K_v_7.3); EEIE7 (in K_v_7.2); RCD3B (in K_v_8.2); FHM3, SMEI, and GEFS^+^ (in Na_v_1.1); and FHM1, EA2 and SCA6 (in Ca_v_2.1). Mutations in *SCN1A* are from a database available at http://www.molgen.ua.ac.be/SCN1AMutations/home/Default.cfm.

K_v_1.1 and K_v_1.2 subunits, belonging to the K_v_1 family of VGKCs, generate delayed rectifier channels active at or below the RMP, with fast activation/deactivation kinetics and slow inactivation, mainly contributing to transient, slowly inactivating current (I_D_). K_v_1 family members are expressed throughout many regions of the central nervous system (CNS), including hippocampus, cerebellum, and brainstem nuclei. In several brain regions, co-assembling of K_v_1.1 with K_v_1.4 subunits, which contain N-terminal Inactivation Modules, confers N-type inactivating properties to heteromeric channels. At neuronal subcellular level, K_v_1.1, K_v_1.2 and K_v_1.4 are expressed in the axon initial segment (AIS) where they control AP threshold and firing (Figure 2), in juxtaparanodal regions next to Ranvier’s nodes, in synaptic terminals and in proximal dendrites (Trimmer and Rhodes, 2004). The important pathophysiological role of K_v_1.1 channels is underlined by the occurrence of mutations in this channel in patients affected with Episodic Ataxia type 1 [EA1; Online Mendelian Inheritance in Man (OMIM) 160120]. EA1 is a rare, disabling condition with autosomal-dominant inheritance characterized by constant myokymia and dramatic episodes of spastic contractions of head, arms and leg muscles, together with the loss of motor coordination and balance. Moreover, EA1 is associated with an increased incidence of epilepsy. Other features include delayed motor development, cognitive disability, choreoathetosis, and carpal spasms. Disease onset is in childhood or early adolescence (D’Adamo et al., 2014). An altered function of K_v_1.1 channels expressed in the cerebellum may be responsible for ataxia, whereas seizures and cognitive dysfunctions associated with EA1 may be caused by impaired K_v_1.1 channels in the hippocampus. The first description of a family affected by EA1 was in 1975 (VanDyke et al., 1975); subsequent genetic analysis revealed a heterozygous point mutation in the coding sequence of the human gene *KCNA1*, encoding for K_v_1.1 channels, leading to the F184C substitution in the S_1_ segment. Electrophysiological studies demonstrated that this mutation shifts the activation curves of K_v_1.1 channels to more positive voltages, therefore leading to loss-of-function effects (Adelman et al., 1995). Several other K_v_1.1 mutations causing EA1 have been described and they appear to be localized in relevant functional regions of the channels, particularly in the VSM (Figure 3) or in the PM. Overall, functional studies performed on numerous mutant channels revealed that EA1-causing mutations may alter membrane expression or gating processes, such as opening and closure kinetics, voltage-dependence of activation, N- and C-type inactivation. An atypical mechanism by which the V408A pore-mutation affects gating of K_v_1.1 channel has been described by means of voltage-clamp fluorometry and single-channel recordings. This mutation accelerates the outward current decay during depolarization by promoting an “activated-not-open” conformation rather than by increasing C-type inactivation (Peters et al., 2011). Moreover, mutations within the VSM cause EA1 mainly by a reduction of maximal currents, in some cases with dominant-negative effects (as in the case of R167M, C185W, F249I, I262T or R307C mutations; Adelman et al., 1995; Zerr et al., 1998; Graves et al., 2010; Zhu et al., 2012; Tomlinson et al., 2013). These results suggest that EA1-causing mutations induce loss-of-function effects on K_v_1.1 channels, with haploinsufficiency appearing as the most likely pathogenetic mechanism for the disease. Accordingly, the loss of K_v_1.1 channels, obtained either by genetic or pharmacological manipulations, caused alterations in the hippocampal network, which showed more frequent and longer spontaneous sharp waves and high frequency oscillations; moreover, in the CA3 region from K_v_1.1^−/−^ mice, mossy fibers and medial perforant path axons were hyperexcitable and produce greater pre- and post-synaptic responses, also showing a reduced paired-pulse ratios suggestive of an increased neurotransmitter release at these terminals (Simeone et al., 2013).

More recently, four different *de novo* mutations in the *KCNA2* gene, encoding for K_v_1.2 K^+^ channel subunits, have been identified in patients with Epileptic Encephalopathy (EE). Three of these mutations affect conserved residues in the VSM (I263T in S_3_, R297Q and L298F in S_4_) and one in the PM (P405L). Functional studies in heterologous expression systems revealed that channels formed by subunits carrying the I263T or P405L mutations almost completely lost their function, with also dominant-negative effects on wild-type channels; by contrast, the two S_4_ mutations caused a gain of function effect (Syrbe et al., 2015). A K_v_1.2 mutation has been also described in a mouse model of cerebral ataxia (I402T substitution in the S_6_ segment), called Pingu: patch-clamp recordings from cerebellar slices revealed an increased frequency and amplitude of spontaneous GABAergic inhibitory postsynaptic currents and reduced AP firing frequency in Purkinje cells, suggesting that K_v_1.2 mutation-dependent increase in GABA release from basket cells is involved in the loss of motor coordination in Pingu mice (Xie et al., 2010).

#### K_v_3.1 (*KCNC1*) and K_v_3.3 (*KCNC3*) Channel Mutations Cause Progressive Myoclonus Epilepsy (PME) or Spinocerebellar Ataxia Type 13 (SCA13), Respectively

The K_v_3 family of VGKCs comprises four members (from K_v_3.1 to K_v_3.4, encoded by the *KCNC1-KCNC4* genes) that differ from others VGKCs because of a more positive activation threshold (thus, they are identified as “high-threshold channels”) and their fast rate of deactivation upon repolarization. These unique properties specifically enable AP fast repolarization without affecting spike initiation and AP duration (Rudy and McBain, 2001; Figure 2). The expression of K_v_3.1, K_v_3.2 and K_v_3.3 is limited to the CNS, whereas K_v_3.4 is mainly expressed in skeletal muscles and sympathetic neurons, and only weakly expressed in a few neuronal types. In the CNS, K_v_3 channels are mainly expressed in fast-spiking neurons, such as GABAergic interneurons. Accordingly, suppression of K_v_3 currents by pharmacological tools (Erisir et al., 1999) or in knockout mice (Lau et al., 2000) impairs firing properties of fast-spiking neurons, affects neurotransmitter release (Sabatini and Regehr, 1997), and induces cell death (Irie et al., 2014).

Spinocerebellar Ataxias (SCAs) are a very heterogeneous group of autosomal dominant neurological disorders caused by degeneration of the cerebellum and spinal cord. SCAs show a wide range of phenotypes, including cerebellar ataxia, dysarthria, extrapyramidal symptoms, oculomotor disturbance, cognitive impairment, and epilepsy. Most SCAs are caused by trinucleotide repeat expansions, while other forms are caused by point mutations in protein kinases, cytoskeletal and mitochondrial proteins, and proteins regulating intracellular calcium stores, such as beta 3 Spectrin (SPTBN2), Tau Tubulin Kinase 2 (TTBK2), Protein Kinase C Gamma type (PRKCG), Inositol 1,4,5-Trisphosphate Receptor type 1 (ITPR1), ATPase Family Gene 3 Like 2 (AFG3L2). In particular, SCA13 (OMIM 605259) is caused by point mutations in the *KCNC3* gene, encoding for K_v_3.3 channels. So far, three mutations have been identified in K_v_3.3, two of which affect residues in the VSM (R420H and R423H both in S_4_; Figure 3) and one in the PM (F448L; Waters et al., 2006; Figueroa et al., 2010). Functional studies performed in heterologous expression systems revealed that both VSM mutations caused a complete abolishment of channel function when expressed homomerically, whereas the co-expression of mutant subunits with wild-type subunits caused a significant suppression of K_v_3.3 activity, consistent with dominant-negative effects. By contrast, the pore mutant F448L channels did not affect current amplitude, but causes a hyperpolarization shift of the current activation and slows the rate of deactivation, suggesting that this mutation increases the stability of the open state (Waters et al., 2006). Taken together, these data suggest that mutations in KCNC3 may cause SCA both by gain- and loss-of-function mechanisms (Figueroa et al., 2010).

More recently, a recurrent mutation in the *KCNC1* gene, encoding for K_v_3.1 channels, has been identified in patients with Progressive Myoclonus Epilepsy (PME7; OMIM 616187; Muona et al., 2015), one of the most devastating form of epilepsy, characterized by a very wide clinical and genetic heterogeneity (Berkovic et al., 1986). Most of PME cases are autosomal recessive, although in few cases an autosomal dominat transmission occurs. Clinically, the most common features of this disease are myoclonus, tonic-clonic seizures and progressive neurological decline. The mutation occurring in K_v_3.1 channel causing PME affects a highly conserved residue in the S_4_ segment (R320H; Figure 3). Functional experiments in heterologous expression systems showed that the R320H substitution caused a prominent loss of function, with dominant-negative effects on wild-type K_v_3.1 channels.

#### K_v_7.2/7.3 (*KCNQ2/KCNQ3*) and Benign Familial Neonatal Seizures (BFNS)/Epileptic Encephalopathy (EE)

K_v_7.2 and K_v_7.3 channels belong to the *KCNQ* gene family that comprises five members (*KCNQ1-5*) encoding for K^+^ channel subunits showing different tissue distribution and physiological roles (Soldovieri et al., 2011). When heterologously expressed, homomeric K_v_7.2 channels generate K^+^-selective currents activated by depolarization at membrane potentials around −50 mV with slow activation and deactivation kinetics and lacking significant inactivation. Currents carried by K_v_7.3 homomultimers are rather small and activate around −60 mV; among K_v_7 members, K_v_7.3 channels show the highest opening probability and unitary conductance at the single-channel level (Li et al., 2005). Co-expression of K_v_7.2 and K_v_7.3 channels generate currents 10-times larger than those obtained by the simple sum of the currents produced by K_v_7.2 or K_v_7.3 homomultimers (Wang et al., 1998; Yang et al., 1998). This effect is mainly mediated by a higher opening probability of K_v_7.2/K_v_7.3 heteromers compared to K_v_7.2 homomers, together with a two- to threefold increase in the number of channel-forming subunits expressed at the plasma membrane (Schwake et al., 2000). In the brain, heteromeric assembly of K_v_7.2 and K_v_7.3 subunits underlies the M-current (I_KM_), a slowly activating and deactivating K^+^ current that regulates neuronal excitability in the sub-threshold range for AP generation (Soldovieri et al., 2011; Figure 2). Mutations in *KCNQ2* (K_v_7.2) and *KCNQ3* (K_v_7.3) genes are responsible for neonatal-onset epileptic diseases with widely-diverging clinical manifestations, ranging from benign to very dramatic phenotypes. Indeed, mutations in these two genes have been identified in patients affected with Benign Familial Neonatal Seizures (BFNS1; OMIM 121200), an autosomal dominant epilepsy of newborns (Biervert et al., 1998; Charlier et al., 1998; Singh et al., 1998), characterized by recurrent seizures that begin in the first days of life and remit after a few weeks or months, with mostly normal interictal EEG, neuroimaging, and psychomotor development (Bellini et al., 2010). In addition, *de novo* missense K_v_7.2 mutations have been recently found in neonates affected with pharmacoresistant seizures, distinct EEG and neuroradiological features, and various degrees of developmental delay, defining a “K_v_7.2 encephalopathy” (Weckhuysen et al., 2012; EIEE7; OMIM 613720). Subsequently, *de novo* missense K_v_7.2 mutations have been also shown as one of the most common cause of early-onset EEs, including the Ohtahara syndrome (Saitsu et al., 2012; Kato et al., 2013), the most severe and earliest developing age-related EE. Most disease-causing K_v_7.2 mutations cluster in the VSM (Figure 3), in the PM or in the long C-terminus of the channel. Functional studies for a significant fraction of the described K_v_7.2 or K_v_7.3 mutations have been carried out, with consequences ranging from slight changes in channel behavior to a complete ablation of channel function (Castaldo et al., 2002; Soldovieri et al., 2006; Miceli et al., 2009). A slight (about 25%) decrease of I_KM_ is believed to be sufficient to cause BFNS, and haploinsufficiency seems to be the primary pathogenetic mechanism for BFNS. Moreover, two independent studies suggested that the clinical disease severity may be related to the extent of mutation-induced functional K^+^ channel impairment (Miceli et al., 2013; Orhan et al., 2014).

BFNS-causing mutations have been also found to neutralize some of the positively-charged residues in the S_4_ segment (R207W, R213W, or R214W; Miceli et al., 2008; Bellini et al., 2010): the functional characterization of K_v_7.2 channels carrying each of these mutations suggests a reduced channel sensitivity to depolarization; accordingly, homology model of K_v_7.2 based on the crystal structure of K_v_1.2/K_v_2.1 chimeric channels revealed that these residues form intrasubunit ionized hydrogen bonds with negatively-charged residues present in the outer cluster which stabilize the activated state of the voltage sensor. Thus, it seems plausible to hypothesize that in mutant channels the activated configuration of the VSM is destabilized, explaining the positive shift in steady-state voltage dependence of activations and possibly the neuronal hyperexcitability in individuals carrying these mutations (Miceli et al., 2008, 2013).

Notably, although most epilepsy-causing mutations in K_v_7.2 or K_v_7.3 so far indentified induced loss-of-function effects, electrophysiological experiments performed on K_v_7 channels carrying *de novo* mutations recently found in individuals affected with various forms of early-onset EEs (R144Q, R201C, or R201H in K_v_7.2, and R230C in K_v_7.3; Rauch et al., 2012; Allen et al., 2013; Carvill et al., 2013; Weckhuysen et al., 2013) have demonstrated that all four mutations cause hyperpolarization shifts of the current activation process by destabilizing the resting state of the VSM (Miceli et al., 2015). Accordingly, computational modeling of a feedforward inhibitory microcircuit formed by a principal hyppocampal CA1 neuron and an inhibitory interneuron suggest that a gain-of-function mutation in K_v_7.2/3-I_KM_ can increase the (apparent) excitability of hippocampal CA1 pyramidal neurons by selective suppression of interneuron activity. These data suggest that K_v_7.2/3 mutations may cause human epilepsy by both loss-of-function and gain-of function mechanisms; whether these two mechanisms underlie different clinical entities (in terms of age of onset, severity, prognosis, and pharmacosensitivity) is yet unknown.

#### K_v_8.2 (*KCNV2*) Mutations Cause Cone Dystrophy with Supernormal Rod Electroretinogram (CDSRR) or Epilepsy

K_v_8.2 subunits are structurally similar to others VGKCs, although they do not form functional channels in homomeric configuration: for this reason, they are also called “silent modulators”. Conversely, they are functional upon assembly in heteromeric channels with other subunits (e.g., K_v_2). The *KCNV2* gene encoding for K_v_8.2 subunits was first cloned from human testis cDNA, but its expression has been also detected in other tissues, such as the photoreceptor layer of the human retina (Wu et al., 2006) and in the hippocampus, where it co-localizes with K_v_2.1 (Allen Institute for Brain Science^1^), the major contributor for delayed rectifier K^+^ currents in neurons (Murakoshi and Trimmer, 1999). Co-assembling of silent K_v_8.2 with K_v_2.1 subunits causes a decrease in the maximal currents as well as modifications in the activation, inactivation, and deactivation kinetics (Bocksteins and Snyders, 2012). Mutations in K_v_8.2 channels, some of them located within the VSM (Figure 3), cause cone dystrophy with supernormal rod response (RCD3B; OMIM 610356), a very rare autosomal recessive retinal disorder, characterized by reduced visual acuity, photoaversion, night blindness, and abnormal color vision. At an early age, the retina shows subtle depigmentation of the macula and, later, more obvious areas of atrophy. Electroretinographic features are characteristic and essential for diagnostic purposes. In addition to RCD3B, two mutations in K_v_8.2 channels have been identified in two unrelated children affected by epilepsy: one mutation is located in the N-terminal region (R7K), and the other in the S_1_ segment of the VSM (M285R; Figure 3). Functional studies demonstrated that both mutant channels increased K_v_8.2-mediated suppression of K_v_2.1 currents. Moreover, heteromeric channels incorporating M285R mutant subunits exhibited additional gating defects, including a +10 mV shift in the voltage dependence of activation and slower activation kinetics. Taken together, these data suggest that both variants may decrease delayed rectifier K^+^ currents in neurons, possibly leading to an increased excitability under conditions of repetitive stimulation (Jorge et al., 2011).

#### K_v_10.2 (*KCNH5*) and Epileptic Encephalopathy (EE)

The *KCNH5* gene encodes for K_v_10.1 potassium channels belonging to the ether-a-go-go (EAG) family (Saganich et al., 1999). EAG family comprises two channel subtypes (K_v_10.1 and K_v_10.2), that are over-expressed in cancer cells (Camacho, 2006). K_v_10.2 is also expressed in the CNS (Saganich et al., 1999), although its function is still unclear (Wulff et al., 2009). Recently, by using whole-exome sequencing in children with sporadic cases of EE of unknown etiology, a novel variant in *KCNH5* has been identified (R327H in S_4_). Structural and functional analysis showed that this pathogenic variant destabilized the resting state of the VSM, therefore leading to a hyperpolarizing shift of the activation curve, suggesting a gain-of-function pathogenetic mechanism associated to this mutation (Yang et al., 2013).

#### K_Ca_4.1 (*KCNT1*) Mutations Cause Malignant Migrating Partial Seizures of Infancy (MMPSI) and Autosomal Dominant Nocturnal Frontal Lobe Epilepsy (ADNFLE)

The *KCNT1* gene encodes for K_Ca_4.1, a sodium-activated potassium channel, also known as “sequence like a calcium activated potassium channel” (Slack) or Slo2.2. Structurally, this channel is similar to the classic VGKCs except for two properties: no charged residues in S_4_ and a very large cytoplasmic C-terminal region. K_Ca_4.1 is highly expressed in different regions of the mammalian brain where it generates a delayed outward current termed I_KNa_ that regulates neuronal excitability. Mutations in *KCNT1* gene have been identified in patients with two different type of epilepsy occurring in infancy or childhood: Malignant Migrating Partial Seizures of Infancy (MMPSI; also called Epilepsy of Infancy with Migrating Focal Seizures, EIMFS) and Autosomal Dominant Nocturnal Frontal Lobe Epilepsy (ADNFLE; Barcia et al., 2012; Heron et al., 2012). MMPSI is a severe early onset epileptic encephalopathy beginning before of 6 months of age. It is characterized by heterogeneous focal seizures, with seizures foci migrating from one brain region and hemisphere to another, and is associated with arrest or regression of development, resulting in profound disability. By contrast, ADNFLE is characterized by nocturnal frontal lobe seizures beginning in mid childhood, with psychiatric, behavioral, and cognitive disabilities in some cases. All the described mutations causing MMPSI and ADNFLE are not located into the VSM; however, they dramatically affect channel function, with a striking gain-of-function phenotype *in vitro* (Milligan et al., 2014).

### Sodium Channelopathies

#### Structure and Function of Voltage-Gated Sodium Channels (VGNCs)

VGNCs contribute to the generation of both somatodendritic and axonal AP and conduct subthreshold persistent Na^+^ currents following AP. Structurally, they are complexes of a pore-forming α subunit of 260 kDa, associated to auxiliary β subunits (β_1_–β_4_) of 33-36 kDa (Figure 1; Catterall, 2000). Auxiliary β subunits can assemble with α-subunits and modify their subcellular localization and biophysical properties. In particular, β_2_ and β_4_ subunits form disulfide bonds with α subunits, whereas β_1_ and β_3_ subunits interact with α-subunits non covalently. Structurally, β subunits are formed by an N-terminal extracellular immunoglobulin-like fold, a single transmembrane α-helix and a short intracellular segment (Isom et al., 1992, 1995).

Each α subunit contains four internally repeated domains (called from DI to DIV), each including a VSM and a PM. In the VSM, S_4_ segments in all four domains have previously-described primary sequence and functional role (Catterall, 1986; Guy and Seetharamulu, 1986; Yarov-Yarovoy et al., 2006, 2012). Furthermore, the DIII-DIV linker and particularly the key IFM motif, plays a crucial role in channel fast inactivation, a process that contributes to the neuronal excitability control.

In humans, 10 genes encode for VGNC α subunits; these are expressed in different excitable tissues and give rise to currents with peculiar biophysical properties (Goldin, 2001). Na_v_1.1, Na_v_1.2, Na_v_1.3 and Na_v_1.6 channel subtypes are the primary Na^+^ channels in the CNS; by contrast, Na_v_1.7, Na_v_1.8 and Na_v_1.9 channel subtypes are mainly expressed in the peripheral nervous system (PNS); finally, Na_v_1.4 channels are expressed in skeletal muscle and Na_v_1.5 in the heart. The tenth sodium channel protein (Na_v_1.10) is not voltage-gated and is involved in salt sensing (Watanabe et al., 2000). At the subcellular level, Na_v_1.1 and Na_v_1.3 channels are primarily localized in cell bodies (Westenbroek et al., 1989, 1992), whereas Na_v_1.2 channels are positioned in unmyelinated or pre-myelinated axons and dendrites, and Na_v_1.6 channels in myelinated axons and dendrites (Caldwell et al., 2000). In the developing neocortex, Na_v_1.1 channels appear to be abundantly expressed in the proximal part of the AIS of parvalbumin- (Ogiwara et al., 2007; Li et al., 2014b) and, at lower levels, somatostatin-positive GABAergic interneurons (Li et al., 2014b). By contrast, in the same region, Na_v_1.2 channels are preferentially distributed to the somatostatin-positive population of GABAergic neurons, whereas Na_v_1.6 channels are mainly located at the distal part of the AIS of both types of interneurons (Li et al., 2014b). Such differences in the distribution of specific Na_v_ channel subtypes appear to explain their differential role in controlling intrinsic excitability of distinct neuronal populations.

A large number of genetic diseases are caused by mutations in VGNCs, including inherited forms of periodic paralysis, cardiac arrhythmia, epilepsy, and chronic pain (Lehmann-Horn and Jurkat-Rott, 1999; Catterall et al., 2008). Among these, the genes most frequently associated with inherited forms of neurological disorders encode for brain Na^+^ channels Na_v_1.1 (*SCN1A*) and Na_v_1.2 (*SCN2A*).

#### Na_v_1.1 and Epilepsy

Mutations in *SCN1A* (Na_v_1.1) are responsible for genetic epilepsy syndromes with a wide range of severity. In fact, *SCN1A*-associated epilepsies range from simple febrile seizures, to Generalized Epilepsy with Febrile Seizures (GEFS^+^; OMIM 604233; Escayg et al., 2000), an autosomal dominant epilepsy disorder associated to missense mutations, to Severe Myoclonic Epilepsy of Infancy (SMEI or Dravet syndrome, OMIM 607208; Dravet et al., 1992; Engel and International League Against Epilepsy [ILAE], 2001), a much more severe form of epilepsy often caused by mutations causing truncation or deletions of Na_v_1.1 channels. SMEI is a drug-resistant epilepsy occurring in the first year of life with seizures often associated with elevated body temperature and progressing to prolonged, clustered, or continuous seizures and to status epilepticus (Dravet et al., 1992; Engel and International League Against Epilepsy [ILAE], 2001).

Although only few of the Na_v_1.1 mutations responsible for epileptogenic diseases in humans have been functionally characterized, several evidence point to a loss-of-function as their common effect (Bechi et al., 2015), with a correlation between the increasing functional severity of Na_v_1.1 channel alteration and the worsening of the clinical phenotype (Catterall et al., 2010). In other words, missense mutations found in patients with more benign clinical courses cause milder channel dysfunction, whereas more dramatic functional consequences are caused by truncation or loss-of-function mutations described in more severe phenotypes. Disease-causing Na_v_1.1 mutations appear to be spread along the α subunit sequence, with 25% of those (157/675) affecting the VSM.^2^ However, other studies (Kanai et al., 2004) have reported the occurrence of Na_v_1.1 missense mutations in SMEI affecting the PM, suggesting that, beside the type of mutations, also their localization plays a part in the expressed phenotype. Finally, in some cases mutations of the same residue, but introducing different amino acids, have been identified in patients with SMEI (R1648C) or GEFS^+^ (R1648H; see below), suggesting that the specific structural and functional changes prompted by each mutation are strictly related to the severity of the disease. One of the possible molecular mechanism responsible for the loss-of-function occurring in Na_v_1.1 disease-causing mutations is a folding defect, whereby the quality control system of the endoplasmic reticulum recognizes the mutation-induced structural defect and targets the abnormal protein to degradation, severely impeding its membrane trafficking. Therefore, rescuing defective proteins from degradation appears as an attractive intervention strategy in Na_v_1.1-related disease. Among the various strategies to reverse Na_v_1.1 folding defects, co-expression of β_1_ subunits (Sugiura et al., 2012), exposure to (lower) temperatures which facilitate correct folding (Bernier et al., 2004; Rusconi et al., 2007), co-expression with modulatory proteins (such as calmodulin; Rusconi et al., 2007), as well as the exposure to Na_v_ blockers (such as phenytoin; Bechi et al., 2015), are the most studied. In fact, the function of the epileptogenic missense mutations, including the R859C affecting the first arginine residue in the DII-S_4_, can be rescued by co-expression of the β_1_ subunit, of the interacting protein ankyrin, and of a modified scorpion toxin binding to the VSM of these channels specifically engineered to target the endoplasmic reticulum (Bechi et al., 2015). Notably, some Na_v_1.1 mutations identified in patients affected with GEFS^+^ or SMEI introduce different substitutions at the same residue; in these cases, the extent of the folding defect appears to correlate with disease severity.

As previously indicated, more than half of the SMEI mutations cause loss-of-function effects due to stop codons or deletions, demonstrating that haploinsufficiency of *SCN1A* is pathogenic. To understand this counterintuitive result, as reduced Na^+^ currents should lead to hypoexcitability rather than hyperexcitability, mouse models were generated by targeted deletion in *SCN1A* gene: notably, the loss of Na_v_1.1 channels substantially reduced Na^+^ currents only in hippocampal inhibitory interneurons of both Na_v_1.1^+/−^ and Na_v_1.1^−/−^ mice, but not in excitatory pyramidal neurons (Yu et al., 2006; Ogiwara et al., 2007), therefore causing a loss of sustained high-frequency firing in hippocampal and cortical interneurons, possibly explaining the hyperexcitability observed in patients affected by SMEI.

Alterations of GABAergic interneurons in Na_v_1.1^−/−^ mice could also contribute to explain additional clinical manifestations occurring in SMEI patients: in fact, Na_v_1.1^+/−^ mice have a reduced non-REM sleep, impaired circadian behavior, cognitive impairment and autistic-like traits, reminiscent of those observed in children with SMEI (Han et al., 2012).

Although the functional consequences of SMEI-associated mutations are generally recapitulated in *SCN1A* knockout mice, functional studies in heterologous expression systems of Na_v_1.1 channels incorporating GEFS^+^-associated mutations suggest that both loss- and gain-of-function effects may contribute to the pathogenesis of this disease. In particular, functional studies in an animal model of GEFS^+^ incorporating the R1648H mutation affecting the S_4_ in domain IV of Na_v_1.1 channels (Figure 3; Tang et al., 2009; Martin et al., 2010) have suggested that this variant reduced currents in both excitatory and inhibitory neurons, although in a different way: indeed, this variant reduced peak Na^+^ currents and enhanced slow inactivation in inhibitory neurons (possibly leading to hypoexcitability of inhibitory neurons), whereas it negatively shifted the voltage dependence of fast inactivation in excitatory neurons (possibly leading to hyperexcitability of excitatory neurons). As similarly observed for SMEI-model mice, the combination of these two mechanisms lead to a predominant reduction *in vivo* of GABAergic neurons excitability, therefore explaining the hyperexcitability observed in GEFS^+^-affected patients. More recently, a comprehensive neurophysiological analysis of this mouse model has confirmed that the R1648H mutation reduces firing in inhibitory neurons from various brain regions, but does not affect excitability of excitatory neurons (Hedrich et al., 2014).

#### Na_v_1.1 Channels and Familial Hemiplegic Migraine (FHM3)

Familial Hemiplegic Migraine (FHM) is a rare, monogenic subtype of migraine with aura, associated to mutations in three genes. In particular, FHM1 (OMIM 141500) is caused by mutations in the CACNA1A calcium channel subunit (see below), FHM2 is caused by mutations in the α2 subunit of the Na, K-ATPase pump (ATP1A2; OMIM 602481), and FHM3 has been more recently associated to mutations in Na_v_1.1 channels (de Vries et al., 2009; OMIM 609634). All these proteins are crucial regulators of ion fluxes across neuronal and glial cell membranes, suggesting that FHM, and possibly other forms of migraine, should be considered as cerebral ionopathies.

So far, only six FHM3-causing mutations have been identified: one of them (L1649Q) affects the DIV-S_4_ segment at the level of a residue following R1648, which is mutated both in SMEI- or GEFS^+^-affected patients. Other FHM3-causing mutations are present in the pore (L263V) or in the DIII-DIV linker (Q1489K/H and F1499L). Finally, one mutation in the intracellular DII-DIII linker (T1174S) has been recently found in a family affected by FHM and epilepsy. Complex alterations prompted by this mutation on the functional properties of Na_v_1.1 channels, including loss- (such as a rightward shift in the activation curve and a slower recovery from fast inactivation) and gain- (increased persistent sodium currents, I_NaP_) of-function mechanisms each appear to be involved in the proepiletogenic and pro-FHM manifestations of the disease, respectively (Cestèle et al., 2013a).

The functional consequences prompted by the VSM-affecting mutation L1649Q were first assessed by introducing this mutation in the highly homologous human Na_v_1.5 (encoded by the *SCN5A* gene), revealing a slower inactivation and a faster recovery from inactivation, predicting enhanced neuronal excitation (Vanmolkot et al., 2007). However, a subsequent study, in which this mutation was introduced in Na_v_1.1 channels and co-expressed with both human β_1_ and β_2_ accessory subunits (Kahlig et al., 2008), revealed a complete loss of function, possibly because of a markedly reduced membrane expression of Na_v_1.1-L1649Q mutant channels. Notably, a subsequent paper has demonstrated that the exposure of transfected cells to permissive temperature (30°C for 36–48 h) before recordings, as well as the expression of this mutant in neurons at physiological temperature (37°C), was able to partially restore the function of Na_v_1.1-L1649Q channels, which therefore behaved like folding defective mutants; strikingly, rescued currents showed altered biophysical properties consistent with gain-of-function effects on neuronal hyperexcitability (Cestèle et al., 2013b).

Gain-of-function effects are also caused by the pore-affecting mutation L263V (Kahlig et al., 2008); by contrast, the Q1489K mutation appears to be associated to complex functional alterations, particularly in the inactivation process, leading to predominant loss-of-function effects when expressed in Na_v_1.5 (Vanmolkot et al., 2007) or in the long isoform of Na_v_1.1 (Kahlig et al., 2008) channels, rather than to predominant gain-of-function when introduced in the shorter isoform of Na_v_1.1 (the predominant variant expressed in the brain; Schaller et al., 1992) and studied by transfections of heterologous cells or cultured neurons (Cestèle et al., 2008).

Two additional Na_v_1.1 mutations not affecting the VSM but rather the DIII-DIV linker (Q1498H and F1499L) can also cause Elicited Repetitive Daily Blindness (ERDB), suggesting that *SCN1A* mutations can cause a complex spectrum of human disease, including epilepsy, FHM3 and/or retinal cell excitability (Vahedi et al., 2009).

#### Na_v_1.2 and Benign Familial Neonatal-Infantile Seizures (BFNIS)

Benign Familial Neonatal-Infantile Seizures (BFNIS or BFIS3; OMIM 607745) is a mild seizure syndrome caused by dominant mutations in *SCN2A* encoding for Na_v_1.2 channels (Heron et al., 2002). Affected individuals have seizures starting in early infancy (Berkovic et al., 2004). Typically, ictal episodes begin as partial seizures, which often become generalized. Febrile seizures are rare. Fortunately, these seizures favorably respond to treatment with anti-epileptic drugs, and they generally remit by 1 year of age. Na_v_1.2 channels are closely related to Na_v_1.1 channels in amino acid sequence and in their CNS expression pattern (Goldin et al., 2000). They are primarily expressed in unmyelinated axons in adult rodent brain (Westenbroek et al., 1989), being replaced by Na_v_1.6 channels during axon myelination (Rasband, 2010). Na_v_1.2 expression precedes that of Na_v_1.1, also increasing during the first 4 weeks of postnatal life in rodents (Gordon et al., 1987; Beckh et al., 1989). The relationship between the functional alterations in Na_v_1.2 channels and the hyperexcitability of BFNIS is not yet clear, also because not all BFNIS-causing mutations have been functionally characterized and because contrasting results have been obtained in some cases upon expression of the same mutant channels in different expression systems. However, for many VSM-affecting mutations, such as R223Q (in the DI-S_4_) and R1319Q (DIII-S_4_) gain-of-function effects have been reported (Sugawara et al., 2001; Scalmani et al., 2006), through complex biophysical alterations combining a hyperpolarized shift in the voltage-dependence of activation and a positive shift in the voltage-dependence of inactivation: these results suggest an increase in Na^+^ channels availability, leading to hyperexcitability. In addition, a further missense mutation (L1563V, within DIV-S_2_) in a specific Na_v_1.2 splice variant expressed at the neonatal stage caused a gain-of-function effect (Xu et al., 2007). Notably, this neonatal isoform has a more positive voltage-dependence of activation than the adult one, which may contribute to the relative hypoexcitability of the neonatal brain; moreover, since the effect of the mutation was specific for this neonatal variant, it appears likely that seizure disappearance after the neonatal period in BFNIS patients carrying this mutation is due to the switch between the two Na_v_1.2 isoforms or to the developmental switch between Na_v_1.2 and Na_v_1.6 channels (Liao et al., 2010b).

In addition to BFNIS, *de novo* dominant mutations in *SCN2A* can cause also more severe seizure syndromes (Kamiya et al., 2004; Ogiwara et al., 2009; Liao et al., 2010a; EIEE11; OMIM 613721): as recently reviewed (Baasch et al., 2014), 14 mutations in Na_v_1.2 appear to relate with BFNIS and 21 mutations with more severe phenotypes, including developmental delay and intractable seizures. One of the functionally characterized mutations affecting the VSM, namely the *de novo* E1211K mutation, affecting a highly-conserved residue in the DIII-S_1_ and found in a patient affected with intractable seizures and developmental delay, revealed complex alterations in the biophysical properties of Na_v_1.2 channels, including a hyperpolarization of both the half-activation and half-inactivation potentials and a delay in the recovery from inactivation, therefore leading to unpredictable results on mutation-dependent pathogenetic mechanisms. Intriguingly, computational modeling approaches such as those in Cestèle et al. (2013a) could predict the predominant mutation-induced alteration, therefore allowing a better understanding of the pathogenetic mechanisms associated to specific disease-causing mutations.

These results suggest that the spectrum of epilepsies associated to mutations in Na_v_1.2 channels may be similar to that observed for Na_v_1.1 mutations.

#### Na_v_1.3-1.6 Channels and Epilepsy

Recently, pediatric epileptic phenotypes have been also associated to mutations in other two isoforms of the Na_v_1 channel family, namely Na_v_1.3 (*SCN3A*) and Na_v_1.6 (*SCN8A*). Five missense Na_v_1.3 mutations have been identified in patients affected by cryptogenic focal epilepsy, mainly localized in the pore region, except for the D766N mutation, affecting a highly-conserved residue in the S_2_ segment of domain II (Holland et al., 2008; Vanoye et al., 2013). The characterization of the functional consequences prompted by these mutations in Na_v_1.3 channels revealed heterogeneous effects, including smaller current density, slower activation kinetics, depolarized voltage-dependence of activation and inactivation (particularly in the case of the R357Q mutation), or increased persistent currents (as in the case of the E1111K mutation). However, the presence of each of these mutations induces a common functional alteration in Na_v_1.3 currents, consisting in an increased current activation in response to depolarizing voltage ramps, which could contribute to neuronal hyperexcitability (Vanoye et al., 2013).

In addition, mutations in *SCN8A* have been recently identified in patients affected with epilepsy and/or intellectual disability (Larsen et al., 2015; Epileptic Encephalopathy-13, EIEE13; OMIM 614558). The role of this channel in neuronal excitability has been firmly assessed by numerous studies in animal models expressing diverse null *SCN8* variants and revealing that a reduced expression of *SCN8A*-encoded Na_v_1.6 channels protects against seizures by decreasing neuronal excitability (O’Brien and Meisler, 2013). After the identification of the first mutation in the pore of Na_v_1.6 channels in a patient affected with a severe EE (N1768D; Veeramah et al., 2012), other mutations have been identified also in the VSM (R223G in the DI-S_4_, T767I in DII-S_1_, and others; Estacion et al., 2014; de Kovel et al., 2014; Blanchard et al., 2015; Kong et al., 2015). The functional properties of these mutant channels include both loss- and gain-of-function characteristics, revealing also in this case complex pathogenetic mechanisms underlying these severe phenotypes.

#### Na_v_1.7, 1.8, and 1.9 and Pain Disorders

Human genetic studies have clearly revealed a central role for Na_v_1.7, Na_v_1.8, and Na_v_1.9 Na^+^ channels in nociception. In fact, mutations in *SCN9A* (encoding for Na_v_1.7), *SCN10A* (encoding for Na_v_1.8) or *SCN11A* (encoding for Na_v_1.9) occur in many human disorders characterized by an altered pain sensation.

*SCN9A* mutations have been found in families affected by excruciating pain syndromes including Primary Erythermalgia (PE; OMIM 133020), Paroxysmal Extreme Pain Disorder (PEPD; OMIM 167400), or small-fiber neuropathy (OMIM 133020), as well as by Congenital Insensitivity to Pain (CIP; OMIM 243000), an autosomal recessive disorder in which patients are insensitive to pain caused by fractures, burns, dental extractions, and childbirth. Functional characterization of PE-, PEPD- or neuropathy-causing mutations (often occurring in the VSM, as in the case of W1538R in DIV-S_2_ and L823R in DII-S_4_; Lampert et al., 2009; Cregg et al., 2013) revealed gain-of-function effects through different mechanisms (including hyperpolarization in the activation voltage, slowing of the deactivation, increased current size, or impairment of channel inactivation). On the other hand, loss-of-function coding or splicing mutations in *SCN9A* leading to a substantial impairment in dorsal root ganglion (DRG) AP firing have been found in CIP families (Cox et al., 2006).

A wide genetic screen in patients with small-fiber neuropathy and negative for *SCN9A* mutations identified seven disease-causing variants in *SCN10A* (Familial Episodic Pain Syndrome, FEPS2; OMIM 615551). The functional consequences prompted by these mutations have been assessed only for some of them, revealing gain-of-function effects on Na_v_1.8 currents, and hyperexcitability of DRG neurons. None of these mutations has been found to affect the VSM (Faber et al., 2012; Huang et al., 2013; Han et al., 2014).

Other cohorts of patients affected by episodic pain or by small-fiber neuropathy have been also reported to carry mutations in the *SCN11A* gene (Familial Episodic Pain Syndrome type 3, FEPS3; OMIM 615552). Some of these mutations, which also affect the voltage sensing modules, increased Na_v_1.9 current density and enhanced excitability of DRG neurons (Zhang et al., 2013; Huang et al., 2014). On the other hand, a different mutation in *SCN11A* has been also identified in individuals with congenital inability to experience pain who suffer from recurrent tissue damage and severe mutilations (Leipold et al., 2013; Hereditary Sensory and Autonomic Neuropathy type 7, HSAN7; OMIM 615548). This mutation strongly hyperpolarized the voltage-dependence of Na_v_1.9 current activation (a gain-of-function change), while reducing the excitability of DRG neurons expressing the mutant channel (a loss-of-function at the cellular level). The enhanced activity of Na_v_1.9 channels is believed to lead to an inhibition of neuronal firing of DRGs because of a strong depolarization of the RMP which impairs transmission from presynaptic afferents to postsynaptic cells within the spinal cord, thereby producing pain insensitivity (Waxman et al., 2014).

### Calcium Channelopathies

#### Structure and Function of Voltage-Gated Calcium Channels (VGCCs)

VGCCs are involved in a wide range of physiological processes, such as muscle contraction, neurotransmitter release, hormone secretion and gene expression. According to their threshold of activation, VGCCs are classified in two major categories: low voltage-activated (LVA) and high voltage-activated (HVA). Structurally, HVA are heteromultimeric protein complexes formed by co-assembling of a pore forming α_1_ subunit and α_2_, δ, β, γ ancillary subunits (Figure 1); by contrast, LVA channels appear to lack ancillary subunits (Catterall et al., 2005). There are three major families of Ca_v_ α_1_ subunits, namely Ca_v_1, Ca_v_2 and Ca_v_3, each including several members: based on their biophysical and pharmacological features, HVA channels include Ca_v_1 (encoding for L-type channels) and Ca_v_2 (encoding for P/Q-, N- or R-type channels) families, whereas LVA channels comprise the Ca_v_3 family (encoding for T-type channels). The Ca_v_1 channel family encodes three different neuronal L-type channels (called Ca_v_1.2, Ca_v_1.3, and Ca_v_1.4), in addition to a skeletal muscle-specific isoform Ca_v_1.1 channel; the Ca_v_2 channel family includes three members (Ca_v_2.1, Ca_v_2.2, and Ca_v_2.3); among them, Ca_v_2.1 channels gives rise to P- or Q-type channels by alternative splicing and assembly with specific ancillary subunits; Ca_v_2.2 encodes for N-type channels and Ca_v_2.3 for R-type channels. Ca_v_3 channel family includes three members (Ca_v_3.1, Ca_v_3.2 and Ca_v_3.3), corresponding to T-type calcium channels (Simms and Zamponi, 2014).

So far, several human disorders have been linked to mutations in VGCCs affecting the skeletal muscle and the nervous system. In particular, mutations in Ca_v_1.2 are associated to Timothy Syndrome (TS), characterized by severe arrhythmia and multiple organ systems dysfunctions, including neurological disorders such as autism and mental retardation. By contrast, mutations in Ca_v_3.2 channels have been found in various forms of idiopathic generalized epilepsies: disease-associated mutations prompted gain-of-function effects, leading to an increased neuronal excitability and changes in gene transcription (Eckle et al., 2014). To the best of our knowledge, no TS- or epilepsy-causing mutations in Ca_v_1.2 or Ca_v_3.2 genes, respectively, has been found to affect the VSM; thus, these mutations will not be further addressed in the present manuscript. By contrast, mutations in Ca_v_2.1 are associated to migraine (FHM1) and ataxias (EA2 and SCA6); since mutations in Ca_v_2.1 often affect the VSM, these diseases will be discussed in the following section.

#### Ca_v_2.1 Mutations Linked to Familial Hemiplegic Migraine Type 1 (FHM1), Episodic Ataxia Type 2 (EA2) and Spinocerebellar Ataxia Type 6 (SCA6)

Ca_v_2.1 channels are predominantly expressed in neuronal and neuroendocrine cells, where they play a crucial role in neurotransmitter release, particularly at excitatory synapses (Westenbroek et al., 1995). Based on their expression in areas involved in migraine and/or migraine pain pathogenesis, including the cerebral cortex, the trigeminal ganglia, and brainstem nuclei, as well as the cerebellum, it is not surprising that mutations in the *CACNA1A* gene encoding for Ca_v_2.1 have been linked to neurological disorders, such as Familial Hemiplegic Migraine type 1 (FHM1; OMIM 141500), Episodic Ataxia type 2 (EA2; OMIM 108500) and Spinocerebellar Ataxia type 6 (SCA6; OMIM 183086); patients carrying Ca_v_2.1 mutations often present significant overlapping symptoms, suggesting shared pathophysiological mechanisms (Pietrobon, 2013). As previously described for FHM3, FHM1 is a rare, autosomal dominant form of migraine with aura, characterized by recurrent attacks of disabling headache and, in some cases, progressive cerebellar atrophy. So far, 25 mutations in Ca_v_2.1 have been found in FHM1-affected patients (de Vries et al., 2009; Pietrobon, 2013), mostly affecting the pore and the VSM (Figure 3). Functional expression in heterologous systems revealed that a common functional effect of FHM1 mutations is a shift in the activation curve toward hyperpolarized voltages, thus increasing Ca_v_2.1 open probability and leading to gain-of-function effects (Tottene et al., 2002). In particular, the functional effects of the VSM-affecting R192Q mutation have been studied in a knock-in mouse model: electrophysiological recordings from cerebellar granule cells of this mouse showed an increase in Ca_v_2.1 current density, a hyperpolarizing shift in the voltage-dependence of current activation, an increase in calcitonin gene-related peptide (CGRP) release and increased susceptibility to cortical spreading depression (Tottene et al., 2009), a mechanism that is believed to cause the migraine-preceding aura (Lauritzen, 1994). R192Q-induced gain-of-function of Ca_v_2.1 channels appears also responsible for a larger AP-evoked calcium current and a longer AP duration in Capsaicin-Insensitive Trigeminal ganglion neurons (CI-T) when compared to CI-T neurons from WT mice (Fioretti et al., 2011). These effects appear rather specific for glutamatergic neurons, as also the probability of glutamate release appears enhanced in Ca_v_2.1-R192Q expressing neurons (Tottene et al., 2009) when compared to wild-type neurons, whereas gating properties of the Ca_v_2.1 channels expressed in GABAergic interneurons are barely affected (Vecchia et al., 2014).

EA2 is an autosomal dominant, paroxysmal cerebellar disorder, characterized by ataxia, migraine-like symptoms, interictal nystagmus and cerebellar atrophy. More than 20 *CACNA1A* mutations have been linked to EA2: most of them are nonsense (Jen et al., 1999) or missense (Guida et al., 2001; Wappl et al., 2002) mutations. Most nonsense mutations are predicted to truncate Ca_v_2.1 subunits, resulting in loss-of-function effects; furthermore, their expression in heterologous systems together with wild-type channels produces dominant-negative effects (Jeng et al., 2006). In some cases, generalized epilepsy can also be found associated with EA2 (Jouvenceau et al., 2001), as reported for the R1820X nonsense mutation having both loss-of-function and dominant-negative effects on co-expressed wild-type Ca_v_2.1. No EA2-causing mutation has been identified in the VSM.

SCA6 is an autosomal dominant, paroxysmal cerebellar disorder characterized by late-onset, slowly-progressive ataxia and Purkinje neuron degeneration. This disease is associated with a different degree of a CAG repeat expansion (20–33 repeats) at the distal C-terminus of Ca_v_2.1 channels (Ishikawa et al., 1997; Zhuchenko et al., 1997): therefore, also in this case the VSM is not a hot-spot region for disease-causing mutations.

## The VSM as a Molecular Target for Toxins and Syntetic Compounds

VGICs are molecular targets for several classes of drugs, including anticonvulsants, antiarrhythmics, and local anesthetic molecules; most of them recognize a binding site located within the pore region. More recently, the VSM also emerged as a novel pharmacological target for some classes of toxins and synthetic compounds. In VGNCs, three binding sites for neurotoxins have been identified in the VSM (Catterall et al., 2007). Site 3, located in the transmembrane loop between DIV-S_3_ and DIV-S_4_, is targeted by several groups of polypeptide toxins: α-scorpion toxins, sea-anemone toxins, and some spider toxins. These molecules slow or block Na^+^ channel inactivation by inhibiting the conformational changes coupling channel activation to fast inactivation (Rogers et al., 1996). This mechanism, called “voltage-sensor trapping” (Cestèle et al., 1998), may also describe the mode of action of other gating modifier toxins. Site 4, located in the S_1_–S_2_ and S_3_–S_4_ loops of domain II, is recognized by β-scorpion toxins. These toxins induce both a negative shift in the voltage-dependence of Na^+^ channel activation and a reduction in the maximal current. Finally, site 6, probably located near site 3, is occupied by δ-conotoxins that cause a slowing in Na^+^ channel inactivation. Toxin-induced sensor-trapping can also occur in VGCCs (agatoxins from spiders; McDonough, 2007), and in VGKCs of the K_v_2 and K_v_4 (hanatoxins from tarantula; Swartz and MacKinnon, 1997) or K_v_3 and K_v_11 (sea anemone toxins; Diochot and Lazdunski, 2009) subclasses. More recently, a novel molecular variant of AaTXKβ, namely AaTXKβ_(2–64)_, from *Androctonus australis* scorpion venom has been shown to cause a hyperpolarization shift in the voltage-dependence of activation of K_v_7.4 channels (Landoulsi et al., 2013) with a mechanism reminiscent of that described for β-scorpion toxins acting at site 4 of VGNCs (Dutertre and Lewis, 2010).

Besides toxins, also synthetic compounds may recognize a binding site located within the VSM; this pharmacological action has been exploited in some VGNCs and, among VGKCs, in the K_v_7 subfamily. In fact, two compounds (ICA-121431 and PF-04856264) selectively inhibit currents from Na_v_1.1/Na_v_1.3 and Na_v_1.7 channels, respectively, by interacting with a site located at the extracellular ends of the S_2_ and S_3_ transmembrane segments of domain IV, a region that overlaps with the interaction sites for some scorpion and anemone toxins (McCormack et al., 2013). An analog of the analgesic drug diclofenac, called NH29, has been demonstrated to interact with an external groove formed by the interface of S_1_, S_2_, and S_4_ helices of the VSM in K_v_7.2 channels, where it stabilizes the interaction between two conserved residues in S_2_ (E130) and S_4_ (R207). This compound shifts toward the left the activation curve and increases the maximal current of K_v_7.2 channels, thereby acting as a non-toxin gating modifier (Peretz et al., 2010). By contrast, another anolog of diclofenac, called NH17, is able to block K_v_7.2 channels recognizing a binding site in the VSM. Intriguingly, both compounds, NH17 and NH29, bind to the VSM of transient receptor potential vanilloid 1 (TRPV1) channels exerting opposite effects: indeed, they act as an activator and a blocker of TRPV1 currents, respectively. These two compounds also recognize a binding site in the VSM of the proton channel mVSOP (Kornilov et al., 2014). In addition, also another K_v_7.2/3 channel opener, referred as ICA-27243, seems to interact with the VSM (Wickenden et al., 2008), although the specific amino acids involved in drug binding have not been yet identified (Padilla et al., 2009). The compound ztz240, discovered by a screening of 20,000 compounds using rubidium flux combined with atomic absorption spectrometry, increases K_v_7.2 currents by interacting with the F137 residue in the gating charge pathway of K_v_7.2 channels (Gao et al., 2010; Li et al., 2013). Interestingly, the same phenylalanine (F137) in the S_2_ of K_v_7.2 forms the binding site for a previously reported K_v_11.1 activator, NS1643, which also causes a leftward shift in current activation and slows current deactivation of K_v_7.2 channels (Li et al., 2014a).

## Conclusion

Although the general principles of voltage-sensing have been described by purely functional experiments well over 50 years ago, recent progress in apparently distinct fields of investigation ranging from molecular genetics to X-ray crystallography have contributed to the identification of human diseases caused by altered gating of a large number of VGICs, and to the description of the intimate molecular mechanisms of this process at nearly atomic level resolution. In the present work, we have described the diseases associated with mutations in the VSM of neuronal voltage-dependent K^+^, Na^+^, and Ca^2+^ channels, at the same time reporting on the described functional consequences of some of these disease-causing mutations and on the potential pathogenetic mechanisms. In addition, it should be underlined that the study of the functional consequences of naturally-occurring mutations, beside unraveling the potential disease pathogenetic mechanisms, provide unique opportunities to refine our current view of the role played by specific amino acids in channel function; as an example, the opposite functional behavior shown by K_v_7.2 mutations affecting the distal end of S_4_ (causing a marked rightward shift in gating) when compared to those in the proximal end of the same transmembrane segment (which, instead, caused a negative gating shift), provides strong support to the current view of the molecular motions occurring in S_4_ during voltage-dependent gating. By this effort, we took the opportunity to highlight some of the major controversies and pressing issues in the field. Most channelopathies herein described have quite high penetrance and recognize the genetic mutations as a critical event in disease etiology; therefore, whenever possible, we have attempted to highlight genotype-phenotype correlations emerging from the detailed study of the extent of functional derangements found in most commonly used experimental systems. In an era of fast-advancing genomic techniques which allow the identification of a large number of variants in ion channel genes in several human diseases, such studies may contribute to a better definition of genotype-phenotype correlations, assisting in the definition of disease-based criteria to score variant severity, predict their pathogenetic role, and formulate prognostic hypotheses. Such efforts are likely to provide critical data which will be fed into current efforts to standardize and provide guidelines for the interpretation of sequence variants such as those recently provided by the American College of Medical Genetics and Genomics and the Association for Molecular Pathology (Richards et al., 2015). Curation tools such as ClinGen^3^ or ClinVar,^4^ will be instrumental in the interpretation of the association between variants and genetic diseases, and are also likely to standardize phenotyping routines which may result in a more in-depth disease classification. Another crucial issue is the need for better experimental tools (cellular and animal models) to investigate the mechanism(s) by which each specific variant contributes to disease pathogenesis; similar clinical courses are apparently associated with both loss- and gain-of-function effects by specific ion channel variants, highlighting our rudimentary understanding of the molecular steps involved. Finally, given that the subtle structural transitions occurring during gating are likely specific and distinct for each ion channel family and subfamily, we also believe that the VSM is an underestimated target for drug action; we envision that an improved structural knowledge of channel gating may lead to the design of drugs effective on specific VSM states, thereby allowing patient-tailored therapies for rare diseases caused by each VSM mutation. Such drugs could be also of great therapeutic benefit for more common disorders involving changes in ion channel gating.

## Conflict of Interest Statement

The authors declare that the research was conducted in the absence of any commercial or financial relationships that could be construed as a potential conflict of interest.

## Footnotes

^1^http://mouse.brain-map.org

^2^http://www.molgen.ua.ac.be/SCN1AMutations/home/Default.cfm

^3^http://clinicalgenome.org/

^4^http://www.ncbi.nlm.nih.gov/clinvar/
